# An integrated flavoromics and chemometric analysis of the characteristic flavor, chemical basis and flavor wheel of ancient plant ripened pu-erh tea

**DOI:** 10.1016/j.fochx.2025.102278

**Published:** 2025-02-15

**Authors:** Teng Wang, Nianguo Bo, Yiqing Guan, Dihan Yang, Qiuyue Chen, Yanhui Guan, Songzhi Liu, Zhihui Wang, Hongxing Duan, Yan Ma, Ming Zhao

**Affiliations:** a College of Tea Science & College of Food Science and Technology, Yunnan Agricultural University, Kunming, Yunnan 650201, China; b The Key Laboratory of Medicinal Plant Biology of Yunnan Province, National & Local Joint Engineering Research Center on Germplasm Innovation & Utilization of Chinese Medicinal Materials in Southwestern China, Yunnan Agricultural University, Kunming, Yunnan 650201, China; c Pu'er Pushan Tea Co., Ltd, Pu'er, Yunnan 665000, China; d West Yunnan University of Applied Sciences College of Tea (pu'er)Pu'er, Yunnan 665000, China

**Keywords:** Ancient plant ripened pu-erh tea, Characteristic volatile compounds, Flavor wheel, Sensory characteristics, Taste quality

## Abstract

Ripened pu-erh tea derived from sun-dried green tea leaves of ancient tea plants (APRPT) is recognized for its premium quality and unique flavor profile, yet its characteristic flavor chemistry remains insufficiently elucidated. Here, five representative APRPTs were collected and characterized by integrating flavoromics and chemometric approaches. The APRPTs exhibited a thick, reddish infusion color, a mellow taste, and a strong, long-lasting mushroom aroma. Theabrownins, soluble sugars, caffeine, and gallic acid were identified as characteristic taste components and were responsible for the reddish infusion color and mellow taste. Additionally, 39 characteristic volatile compounds were identified in APRPT, and a flavor wheel for APRPT was developed based on 11 aroma types. Notably, 1-octen-3-ol, 6-methyl-5-hepten-2-one, and (E,E)-3,5-octadien-2-one were responsible for the mushroom aroma. These results provide a theoretical foundation for further studies on APRPT quality development.

## Highlights

• Ancient plant ripened pu-erh tea has a strong and long-lasting mushroom aroma.
• Theabrownins, soluble sugar, caffeine, and gallic acid are the characteristic taste components.
• Thirty-nine characteristic volatile compounds with odor activity values >1 were identified.
• 1-octen-3-ol, (E,E)-3,5-octadien-2-one, and 6-methyl-5-hepten-2-one are responsible for the mushroom aroma.

## Graphical abstract

**Figure:**
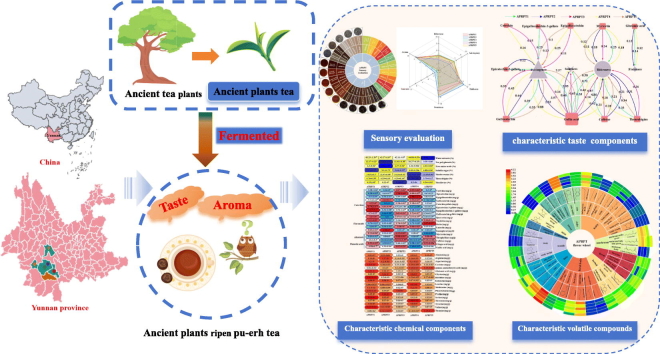

## 1 Introduction

Tea, the second most widely consumed beverage in the world after water, is made from the tender leaves of the tea plant (*Camellia sinensis* (L.) O. *Kuntze*) (Wang, Feng, et al., 2020; Wang, He, et al., 2020; Wang, Yue, & Tong, 2020). It is an important crop that is grown worldwide. In 2022, its cultivation area covered 8,672,730 ha across 48 nations (http://www.fao.org/faostat). Tea plantations in China covered 3,433,100 ha in 2023, making it the largest tea producer in the world. China produces various types of tea, such as green, yellow, black, oolong, dark, and white teas (Mei & Liang, 2024).

Modern tea plants in China and elsewhere are generally pruned to a height of 70 to 80 cm to improve fresh leaf yields and harvesting efficiency. However, there are many unpruned ancient tea plants in China, especially in Yunnan Province. According to the Forestry Industry Standards of the People's Republic of China (LY/T 3311–2022) and (Lu et al., 2021), “ancient tea plants” are defined as tea plants that are at least 100 years old or have a trunk diameter larger than 25 cm. They are not subjected to modern cultivation management or artificial interventions. For example, ancient tea plants are minimally fertilized and not pruned and sprayed with pesticides (Qi et al., 2013). This means that their yields are relatively low. Yunnan Province, situated in the southwestern part of China, is a major tea cultivation area with 513,500 ha of tea plantations in 2023(Mei & Liang, 2024). There are 54,946,700 ancient tea plants in Yunnan Province, which account for 97.70 % of the total number of ancient plants in China (https://www.forestry.gov.cn).

The fresh leaves from ancient tea plants in Yunnan are generally used to produce sun-dried green tea, which is then further processed into ripened pu-erh tea (RPT) through microbial fermentation. Ancient plant ripened pu-erh tea (APRPT) normally commands high prices and has a commercial value due to its extreme scarcity (Pu et al., 2019). Several studies have reported that tea made from ancient plants has a significantly better taste profile and antioxidant activity than other teas (Fang et al., 2024; Ge et al., 2021). A previous study showed that APRPT infusions were much sweeter than other infusions and had a greater soluble sugars content (Wang et al., 2023). Overall, teas made from ancient tea plants have considerable scientific and commercial value, especially those made from APRPT. Currently, APRPT processed from sun-dried green tea of ancient tea mountains such as Laobanzhang in Xishuangbanna, Bingdao, Xigui, and Mengku in Lincang, as well as Kunlunshan, Yangta, Sancaiyun, and Suanzaoshu in Pu'er, have appeared on the market.

The tea drinking experience involves two main elements: taste and aroma. These sensations are intricate and are the result of interactions, enhancements, reductions, and transformations among multiple components rather than simply being the sum of their chemical composition (Wang, Yue, & Tong, 2020). The RPT fermentation process promotes the biotransformation of carbohydrates and phenolic compounds through the action of microorganisms and their enzymes. This process contributes to the mellow taste and stale aroma of RPT. In particular, soluble sugars provide a “sweet and mellow” taste, theabrownin contributes to the "mellow" taste, and volatile compounds, such as methoxybenzene, contribute to the "stale" aroma. They all play significant roles in forming the characteristics associated with RPTs (Wang et al., 2021).

However, as far as can be ascertained, the unique flavor of APRPT and its chemical basis are presently unknown and need to be investigated. Based on this, in this study, five representative APRPTs were collected and their sensory characteristics were evaluated using quantitative descriptive analysis (QDA). The characteristic chemicals and volatile compounds were further investigated using high-performance liquid chromatography (HPLC) and headspace solid-phase microextraction coupled with gas chromatography/mass spectrometry (HS-SPME-GC–MS). Additionally, the dose-over-threshold (DOT) value and the odor activity value (OAV) were combined to identify the key taste compounds and characteristic volatile compounds in APRPT. This information was then used to construct a APRPT flavor wheel. The aim of the study was to scientifically elucidate the quality characteristics of APRPTs and their chemical basis and this information could then be used to help improve its quality.

## 2 Materials and methods

### 2.1 Chemicals and reagents

Reference standards for 18 characteristic compounds were used in this study. A total of eight catechin, six flavonoid, two organic acids, and two alkaloid standards were obtained from Manster biotechnology co., ltd. (Chengdu, China); 18 reference standards for free amino acids were obtained from Agilent Technologies (Beijing, China), and the reference standards for γ-aminobutyric acid and L-theanine were obtained from the National Institutes for food and drug control (Beijing, China). The n-alkane (C7-C40) mixtures were purchased from sigma-Aldrich (St. Louis, MO, USA)

### 2.2 Fermentation and collection of APRPT samples

**Fig. 1:**
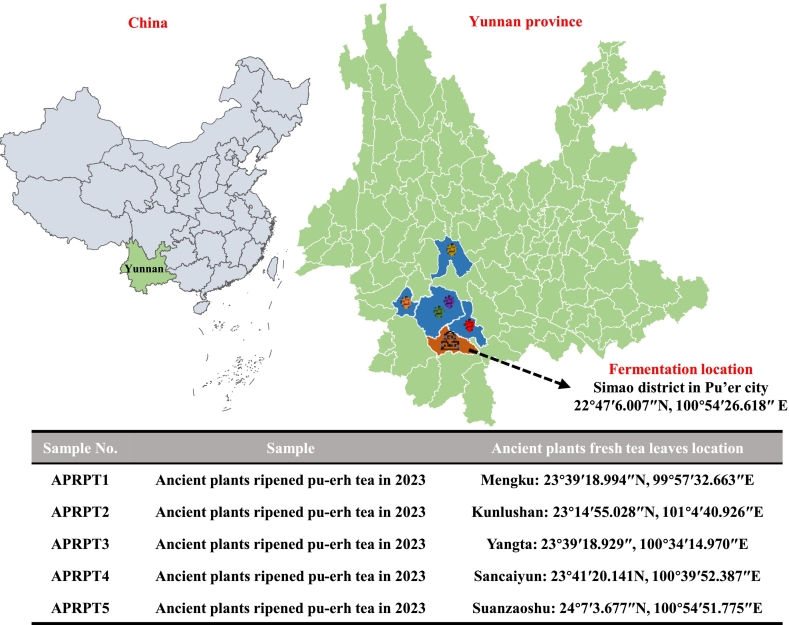
Map showing the locations where ancient plant fresh tea leaves were taken.

Fresh leaves with one bud and three leaves were plucked from ancient tea plantations in Mengku (23°39′18.994″N, 99°57′32.663″E), Kunlushan (23°14′55.028″N, 101°4′40.926″E), Yangta (23°39′18.929″, 100°34′14.970″E), Sancaiyun (23°41′20.141 N, 100°39′52.387″E), and Suanzaoshu (24°7′3.677″N, 100°54′51.775″E), Yunnan, China, in 2023 **(**Fig. 1**)**. The fresh leaves were processed into sun-dried green tea using a traditional method, which included fixing, rolling, and sun-drying to 10 % moisture. Approximately 300 kg of sun-dried green tea from each ancient tea plantation were fermented at Pu'er Pushan Tea Co., Ltd. (22°47′6.007″N, 100°54′26.618″ E), Yunnan, China, between January and August 2023 following traditional methods. Specific steps included adding spring water to the sun-dried green tea leaves to achieve 40 % moisture, piling up the tea to a height of 1 m, and then breaking down the tea pile, mixing it, and re-piling it every seven days. Additional water was added during the re-piling process to maintain 20–30 % moisture. This process was repeated five times and then the leaves were air-dried to 8 % moisture. Samples was collected after the leaves had reached 8 % moisture. Experienced tea makers evaluated all the fermented tea leaves in this study and confirmed that they exhibited the typical characteristics of RPT.

### 2.3 Sensory evaluation of APRPT

A sensory evaluation of APRPT was conducted using the dark tea evaluation method outlined in the Chinese standard (GB/T 23776–2018). The evaluation involved nine trained sensory testers (four males and five females) aged between 24 and 48 years who assessed the appearance, color, aroma, and taste of the infused tea in a specialized sensory evaluation room. The taste of the tea infusion was also evaluated using a QDA, which included its bitterness, astringency, mellowness, sweetness, sourness, and umami (Fan et al., 2021). The taste intensity was evaluated using a scale ranging from 0 to 10, where 0 indicated no perceptible taste and 10 indicated a very strong taste.

### 2.4 Characteristic chemical composition of the tea

The concentrations of the characteristic chemical compounds in the tea leaves were measured using spectrophotometric and HPLC methods. The spectrophotometric method described by Wang et al. (2011) was used to analyze the water extract contents, tea polyphenols, free amino acids, soluble sugars, thearubigins, theaflavins, and theabrownins. Additionally, an Agilent 1200 series HPLC system (Agilent Technologies, Santa Clara, CA, USA) and an Agilent Poroshell 120 EC-C 18 column (4.6 mm × 100 mm, 2.7 μm) were used to separate and measure 18 compounds, including catechins, alkaloids, flavonoids, and phenolic acids (Nian et al., 2019). The concentrations of 18 free amino acids were measured using HPLC with fluorescence detection and an online o-phthalaldehyde (OPA) derivatization method used by (Zhao et al., 2013).

### 2.5 DOT value calculation

A DOT value analysis is frequently used to evaluate the contributions made by specific taste compounds in a taste infusion to the overall taste. The DOT value for each taste compound is calculated by dividing its concentration by its odor threshold (Susanne & Thomas, 2005). The taste attributes and thresholds were sourced from (Susanne & Thomas, 2005) and Fenaroli's Handbook of flavor ingredients (Van Gemert, 2011).

### 2.6 Determination of volatile compounds

The tea samples were ground into a powder using liquid nitrogen and 500 mg was placed in a 20 mL headspace vial (Agilent, Palo Alto, CA, USA). The vial contained a saturated NaCl solution and 20 μL of 10 μg/mL 3-hexanone-2,2,4,4-d4, which served as the internal standard solution.

Each vial was heated to 60 °C for 5 min. Then, a 120 μm DVB/CWR/PDMS fiber (Agilent, Santa Clara, CA, USA) was inserted into the sample headspace for 15 min at 60 °C. Before sampling, the extraction head sampling fiber underwent aging in a fiber conditioning station at a temperature of 250 °C for 5 min.

A Model 8890 GC apparatus (Agilent) was used to desorb the volatile compounds from the fiber coating at the injection port at 250 °C for 5 min in splitless mode. An Agilent Model 8890 GC coupled with a 7000 D MS (Agilent) was used to identify and quantify the volatile compounds. The MS apparatus was equipped with a DB-5MS capillary column (5 % phenyl-polymethylsiloxane, 30 m × 0.25 mm × 0.25 μm), the carrier gas was helium, the linear velocity was 1.2 mL/min, and the temperature of the injector was maintained at 250 °C. The oven temperature was held at 40 °C for 3.5 min, increased to 100 °C at 10 °C/min, then to 180 °C at 7 °C/min, and finally to 280 °C at 25 °C/min. It was maintained at this temperature for 5 min.

The MS measurements were obtained using the electrospray ionization mode with an energy of 70 eV. The temperatures of the quadrupole mass detector, ion source, and transfer line were adjusted to 150 °C, 230 °C, and 280 °C, respectively. The MS analysis was performed in selected ion monitoring (SIM) mode and identification and quantification of volatile compounds were based on a previously described method (Huang et al., 2024).

### 2.7 OAV calculation

An OAV analysis is frequently used to evaluate the contributions made by specific volatile compounds in a volatile infusion to overall taste. The OAV value for each volatile compound is calculated by dividing its concentration by its odor threshold. The odor threshold information was sourced from (Zhai et al., 2022), while the odor descriptions were taken from (Wang et al., 2022; Zhai et al., 2022), and the Good Scents Company database (https://www.thegoodscentscompany.com/search2.html).

### 2.8 Statistical analysis

The characteristic chemical composition measurements were replicated six times and the data are presented as the mean ± standard deviation. A one-way analysis of variance (ANOVA) with a Dunnett's multiple comparison test was used to evaluate the statistical significance of the variations among samples with *p* < 0.05 considered as statistically significant. A heatmap was generated using TBtools software (Toolbox for Biologists; Version 1.082, China).

## 3 Results and discussion

### 3.1 Sensory characteristics of APRPT

**Fig. 2:**
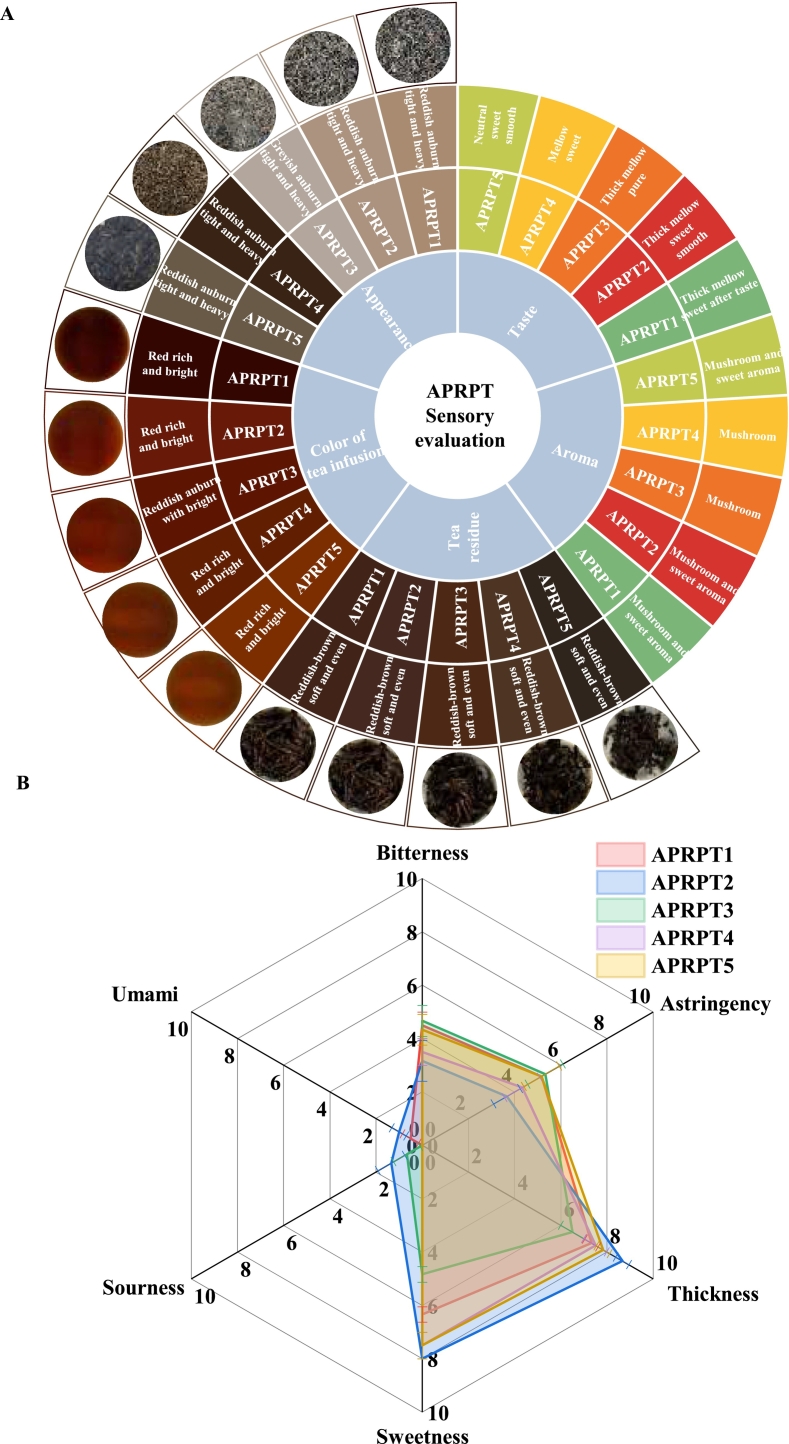
Sensory evaluation of the ancient plant ripened pu-erh tea samples. Sensory attribute wheel (A) and taste sensory scores for the tea infusions (B).

The APRPT teas made by the same tea factory using fresh tea leaves from five famous ancient tea plantations, namely, Mengku, Kunlushan, Yangta, Sancaiyun, and Suanzaoshu, were analyzed **(**Fig. 1**)**. The appearance of the five APRPT samples was tight and heavy, with a reddish auburn color. The tea infusions had a reddish-light color and a mellow taste with a good sweet aftertaste and a long-lasting mushroom aroma. The tea residues were soft and even with a reddish-brown color **(**Fig. 2A**)**. A QDA was used to compare the bitterness, astringency, thickness, sweetness, sourness, and umami of the five APRPT samples. The tea infusion taste scores are shown in Fig. 2B. All the APRPT samples had low sourness and umami scores of 0–1.33. The lowest umami scores were in the range 0–1.00; the lowest bitter and astringency taste scores were in the ranges 3.17–4.67 and 3.67–5.17, respectively; and the strongest sweet and thickness taste scores were in the ranges 5.83–8.00 and 6.50–8.67, respectively. Overall, the five different APRPTs had good sensory qualities and were characterized by a reddish, thick infusion color, a mellow taste, and a strong and long-lasting mushroom aroma.

### 3.2 Characteristic chemical components in the APRPTs

**Fig. 3:**
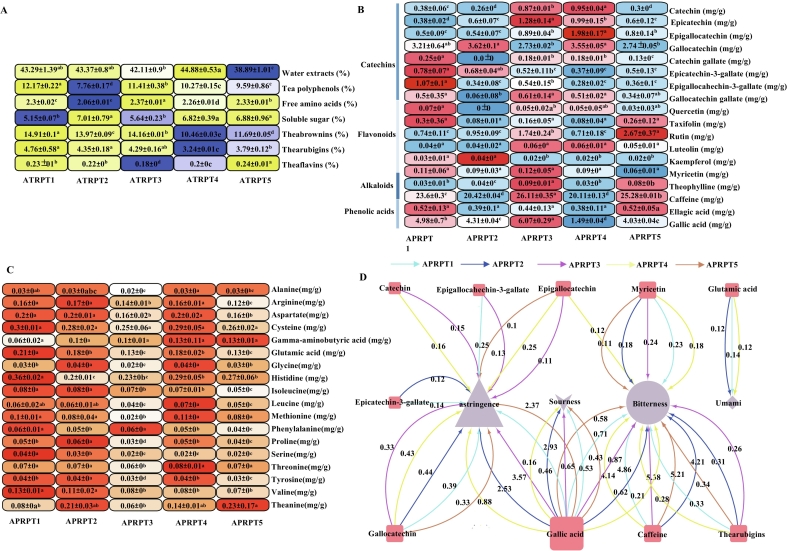
Contents of 43 characteristic compositions that affect the quality characteristics of ancient plant ripened pu-erh tea (A–C). Characteristic compositions (A); catechins, flavonoids, alkaloids, and phenolic acids (B); and free amino acids (C). Network diagram of the taste components (DOT values >0.1) and taste attributes associated with ancient plant ripened pu-erh tea (D). Numbers on the line represent DOT values and the size of the nodes indicates the degree of connectivity.

The taste characteristics of the tea samples, including bitterness, astringency, sourness, sweetness, and umami, are determined by the water-soluble substances in the infusions, such as amino acids, polyphenols, soluble sugars, and tea pigments (Wang, Feng, et al., 2020; Wang, He, et al., 2020; Wang, Yue, & Tong, 2020). In this study, 43 characteristic components were quantitatively measured to determine the components contributing to the “reddish color and mellow taste” of the APRPTs **(**Fig. 3**)**. The water extract content of the APRPT samples ranged from 38.89 % to 44.88 % **(**Fig. 3A**),** which was similar to previous reports (Zhao et al., 2019), and the water extract level was positively correlated with tea quality (Yue et al., 2023) and tea thickness. The total soluble sugar contents varied from 5.15 % to 7.01 %, which was similar to the higher levels of soluble sugars previously reported in APRPT (Wang et al., 2023). Soluble sugar, a sweet substance in tea infusions, helps to moderate bitterness and irritation (Yue et al., 2017) and its levels were positively correlated with mellowness and sweetness (Yue et al., 2017). Among the tea samples, APRPT2 had the largest soluble sugar content at 7.01 % and the highest mellowness and sweetness scores in the sensory evaluation **(**Fig. 3A**)**. The theabrownins content ranged from 10.46 % to 14.91 %, thearubigins ranged from 3.24 % to 4.76 %, and theaflavin ranged from 0.18 % to 0.24 % **(**Fig. 3A**).** Among them, theabrownins are the primary water-soluble pigments and the main color components in tea infusions. They give the infusions a reddish, mellow, and thick infusion color (Deng et al., 2022). Tea polyphenols are a class of polyphenolic compounds primarily composed of catechins. They contribute to the strong astringent and stimulating properties and the bitter and astringent tastes associated with tea infusions (Yue et al., 2017). The tea polyphenol content ranged from 7.76 % to 12.17 %, which was similar to previous reports for tea polyphenol contents in RPT (Wang et al., 2021). The tea polyphenol levels were negatively correlated with bitterness and astringency (Zhou et al., 2023). The APRPT1 sample had the largest tea polyphenol content at 12.17 %, which resulted in the highest bitter and astringency scores **(**Fig. 3A**)**. The microbial fermentation, oxidation, condensation, and degradation of catechins can reduce the catechin content in APRPTs. This process promotes the creation of reddish, rich, and bright infusion colors and a tea that has a mellow, less astringent taste (Lv et al., 2013). Flavonoids have a significant impact on the astringency of tea infusions, but the contents of the six flavonoids investigated in this study were relatively low **(**Fig. 3B**)** and had little influence on the taste of the APRPT samples.

Alkaloids, such as caffeine and theophylline, contribute to the bitter taste of tea. The caffeine content ranged from 20.11 mg/g to 26.11 mg/g and the theophylline content ranged from 0.03 mg/g to 0.09 mg/g **(**Fig. 3B**)**. The caffeine contents were similar to previous reports for RPT (Wang et al., 2021) and the levels were negatively correlated with a strong bitter taste (Lai et al., 2022). The presence of phenolic acids, such as gallic acid and ellagic acid, also contribute to the bitter and sour taste of tea (Zhou et al., 2023). In this study, the gallic acid content ranged from 1.49 mg/g to 6.07 mg/g and ellagic acid ranged from 0.38 mg/g to 0.52 mg/g **(**Fig. 3B**)**.

Free amino acids strongly influence the taste quality of tea infusions. They contribute to the formation of the bitter, astringent, sour, sweet, and umami taste components and also significantly influence the aroma quality of tea (Yu & Yang, 2019). The five APRPT samples were rich in free amino acids, which ranged from 2.06 % to 2.37 % **(**Fig. 3A**)**. A total of 18 free amino acid fractions were quantified. Even though the amino acid content in the APRPTs was low **(**Fig. 3C**)**, it plays a crucial role in complementing and regulating the overall taste of the tea infusion. It is also plays a significant role in the formation of the overall APRPT taste (Yu & Yang, 2019).

### 3.3 DOT values for the taste compounds in APRPT

The taste of an RPT comes from the compounds in the water extract, such as polyphenols and free amino acids. However, it is difficult to accurately assess the impact of these compounds on taste based on their content. A DOT value, calculated using a threshold value, helps characterize the contribution made by different taste compounds, with a DOT value >1 indicating a significant contribution to the taste of the tea infusion (Susanne & Thomas, 2005).

Among the 22 taste compounds that produced a bitter taste, caffeine had DOT values ranging from 4.14 to 5.38 and significantly contributed to the bitter taste of the APRPT samples **(**Fig. 3D**,** Appendix A1**).** This agreed with a previous study, which reported that caffeine is the most abundant alkaloid in tea and is known for its strong bitter taste (Lai et al., 2022). In addition, catechin, gallic acid, and myricetin had 0.1 ≤ DOT value <1, which meant that they actively modified the bitter taste **(**Fig. 3D**,** Appendix A1**)**. Interestingly, all eight bitter amino acids had DOT values of <0.01, indicating that these amino acids did not significantly contribute to the bitter intensity of the tea infusions **(**Appendix A1**)**. Among the 11 astringent taste compounds, the mean DOT value for gallic acid was 2.46, which meant that it significantly contributed to the bitter taste of APRPT **(**Fig. 3D**,** Appendix A1**)**. Previous research has also suggested that gallic acid, a significant phenolic acid in tea, affects the astringency and sourness of the tea infusion (Robichaud & Noble, 2010). Gallocatechin, epicatechingallate, and epigallocatechin gallate had 0.1 ≤ DOT value <1, which meant that they modified astringency, especially the ester catechins **(**Fig. 3D**,** Appendix A1**)**. Previous studies have also shown that ester catechins are important substances that affect the astringency of tea infusions (Zhang et al., 2020). Out of the eight amino acids that contribute to sweetness, seven had DOT values <0.01, which means that they made no significant contribution to taste, but could potentially indicate sweetness. Among the four sour taste compounds, only gallic acid had a DOT value >0.1. The fresh taste in tea is mainly due to glutamic acid, theanine, and aspartic acid. Only glutamic acid had a DOT value >0.1. Theanine and aspartic acid are considered to be potential umami taste substances in APRPTs, but they made no significant contribution in this study **(**Fig. 3D**,** Appendix A1**)**.

Although only two taste compounds, caffeine (bitterness) and gallic acid (astringency), had DOT values >1, the taste of a tea infusion is not solely determined by a single taste property but rather by a combination of many taste substances. There are synergistic and inhibitory effects among them. Some studies have found that certain free amino acids that promote bitterness in tea, such as arginine, tyrosine, and phenylalanine, can enhance the freshness of tea infusions at low levels (Paula et al., 2020). These free amino acids can also decrease the astringency and bitterness caused by caffeine and catechins, thereby enhancing the mellowness and freshness of the tea infusion (Zhang et al., 2017; Zhang et al., 2020).

### 3.4 Volatile compounds in APRPT

**Fig. 4:**
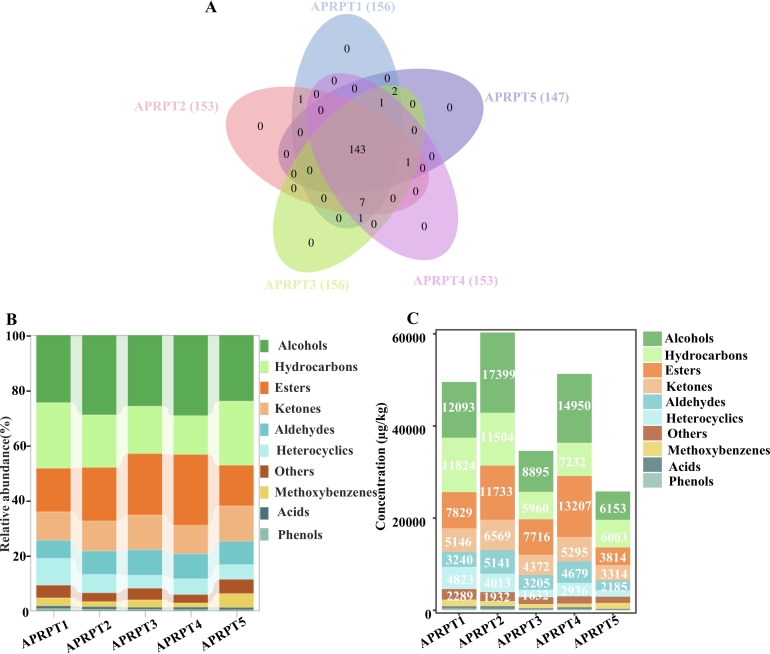
Types and concentrations of volatile compounds in ancient plant ripened pu-erh tea (A–C). Venn diagram showing the number of volatile compounds in each sample (A), relative abundances of the volatile compounds (B), and volatile compound concentrations in the samples (C).

A total of 157 volatile compounds were identified using HS-SPME and GC–MS, and each sample contained 147–156 volatile compounds **(**Figs. 3A–C**,** Appendix A2**)**. These volatile compounds were classified into ten groups based on their chemical structures: hydrocarbons (35 compounds), alcohols (34 compounds), esters (29 compounds), aldehydes (21 compounds), ketones (17 compounds), heterocyclic compounds (6 compounds), acids (4 compounds), methoxybenzenes (4 compounds), phenols (2 compounds), and others (5 compounds) **(**Appendix A2**).** The results were consistent with the classes of volatile compounds in RPT reported by Wang et al. (2022). Both their study and this study suggest that hydrocarbons and alcohols are the most abundant classes of volatile compounds in RPT. The contents of the 157 volatile compounds were measured in the five samples. Alcohols accounted for 23.86 % to 29.14 % of the volatile compounds and were the major volatile compounds found in APRPT. Among them, the (*S*)-(−)-2-methyl-6-methylene-7-octen-4-ol (average concentration: 1863.68 ± 185.36 μg/kg), (6*Z*)-nonen-1-ol (1697.94 ± 172.44 μg/kg), coniferyl alcohol (1589.10 ± 211.81 μg/kg), 1-octen-3-ol (1552.65 ± 120.11 μg/kg), and (E,Z)-2,6-nonadienol (738.63 ± 108.71 μg/kg) contents were relatively high in the APRPT samples **(**Fig. 4B–C**,** Appendix A2**)**.

Esters accounted for 14.79–25.74 % of all volatile compounds, with the most abundant ones being methyl salicylate (4038.01 ± 297.31 μg/kg), (Z)-pent-2-enyl butyrate (1301.39 ± 84.72 μg/kg), dihydroactinidiolide (541.32 ± 31.26 μg/kg), and benzyl isovalerate (378.32 ± 14.56 μg/kg) **(**Fig. 4B–C**,** Appendix A2**)**. Among the esters detected, the average methyl salicylate content was significantly higher than the other esters **(**Appendix A2**)**. Hydrocarbons accounted for 14.10–23.86 % of the volatile compounds, with saturated hydrocarbons, such as perillen, sativene, γ-elemene, α-pinene oxide, α-bisabolene, and γ-terpinene, contributing more to the tea aroma than unsaturated hydrocarbons (Lv et al., 2014). Among the hydrocarbons, the average concentration of cyclosativene was highest at 944.59 ± 119.61 μg/kg, followed by α-funebrene (828.33 ± 25.52 μg/kg). Additionally, perillen (686.90 ± 43.77 μg/kg), sativene (944.59 ± 119.61 μg/kg), γ-elemene (162.04 ± 11.06 μg/kg), α-bisabolene (153.67 ± 13.93 μg/kg), and γ-terpinene (106.33 ± 14.40 μg/kg) **(**Appendix A2**),** were identified in the five samples. Ketones can be produced by Maillard reactions and enzymatic amino acid or lipid reactions by microorganisms (Wang et al., 2019). They make essential contributions to the pu-erh tea aroma (Pang et al., 2019) and 5-methyl-4-hexen-3-one (1031.36 ± 74.97 μg/kg), 3-methyl-2-cyclohexen-1-one (949.19 ± 86.09 μg/kg), β-Ionone (680.33 ± 50.67 μg/kg), (E,E)-3,5-octadien-2-one (548.19 ± 40.05 μg/kg), and isophorone (423.65 ± 41.60 μg/kg) were the most abundant ketones (Appendix A2**)**. Aldehydes are formed through the oxidation and decomposition of lipids (Barra et al., 2005) and accounted for 6.54–9.36 % of the volatile compounds. (Z)-6-nonenal (770.43 ± 57.77 μg/kg), lilac aldehyde C (540.45 ± 40.07 μg/kg), nonanal (511.30 ± 39.69 μg/kg), and (E)-2-nonenal (330.81 ± 21.53 μg/kg) were the most abundant aldehydes **(**Appendix A2**)**. (Ho et al., 2015) reported that phenylacetaldehyde and benzaldehyde have floral aromas and are key volatile compounds that contribute to the creation of the characteristic aroma associated with tea. However, benzaldehyde was not identified in this study, but various benzaldehyde derivatives were identified, such as 4-(1-methylethyl)-benzaldehyde and 4-methylbenzaldehyde **(**Appendix A2**)**. In addition, heterocyclics (4.72 %–9.73 %), methoxybenzenes (1.54 %–5.01 %), acids (0.77 %–0.99 %), and phenols (0.36 %–0.77 %) were found in all the samples at relatively low levels **(**Fig. 4B–C**)**. In brief, the alcohol, hydrocarbon, and ester contents were the highest in all five samples, which was consistent with previous reports (Wang et al., 2022).

### 3.5 Characteristic volatile compounds in APRPT

**Fig. 5:**
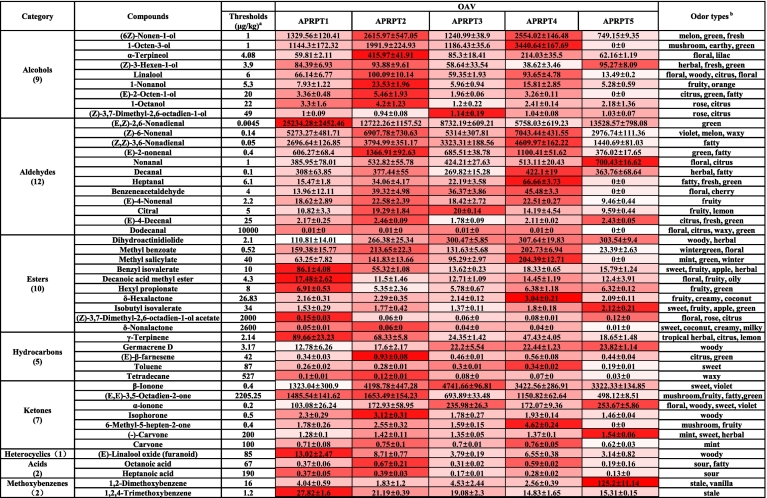
Odor Activity Values (OAV) for the volatile compounds and their odor types. Note: ^a^ The odor threshold was obtained from Zhai et al. (2022). ^b^ The odor types were taken from Wang et al. (2022), Zhai et al. (2022), and the Good Scents Company database (https://www.thegoodscentscompany.com/search2.html).

In the field of flavoromics, the intensity of the aroma contribution made by a volatile compound is typically measured using the OAV. Compounds with an OAV > 1 are usually considered to be characteristic volatile compounds (Ma et al., 2023). Therefore, the OAV can be used to determine the individual contribution made by each volatile compound to the overall aroma. Odor thresholds for 48 volatile compounds were identified and the OAV distribution is shown in Fig. 5. Among them, the OAVs for 39 volatile compounds were greater than one and these were categorized into seven classes, including aldehydes (11 compounds), alcohols (9 compounds), esters (8 compounds), ketones (6 compounds), hydrocarbons (2 compounds), methoxybenzenes (2 compounds) and heterocyclics (1 compound). In general compounds with an OAV > 1 are usually considered to be characteristic volatile compounds (Ma et al., 2023); therefore, these 39 volatile compounds were considered to be characteristic volatile compounds in the APRPT samples.

The OAVs of 17 compounds were greater than 50: (E,Z)-2,6-nonadienal (average OAV = 13,195.07), (Z)-6-nonenal (average OAV = 5503.05), β-ionone (average OAV = 3401.67), (Z,Z)-3,6-nonadienal (average OAV = 3173.12), (6Z)-nonen-1-ol (average OAV = 1697.94), 1-octen-3-ol (average OAV = 1552.65), (E,E)-3,5-octadien-2-one (average OAV = 1096.37), (E)-2-nonenal (average OAV = 827.02), nonanal (average OAV = 511.30), decanal (average OAV = 348.22), dihydroactinidiolide (average OAV = 257.77), α-ionone (average OAV = 187.55), α-terpineol (average OAV = 167.45), methyl benzoate (average OAV = 146.16), methyl salicylate (average OAV = 100.95), (Z)-3-hexen-1-ol (average OAV = 74.16), and linalool (average OAV = 66.54). These compounds may be responsible for the floral fruity, sweet, mushroom, fruity, herbal, woody, floral, mint, and stale aromas associated with the APRPT samples **(**Fig. 5**)**. In summary, the results suggest that the above compounds are characteristic volatile compounds in APRPTs and have an important influence on their aroma.

### 3.6 Flavor wheel for the APRPTs

**Fig. 6:**
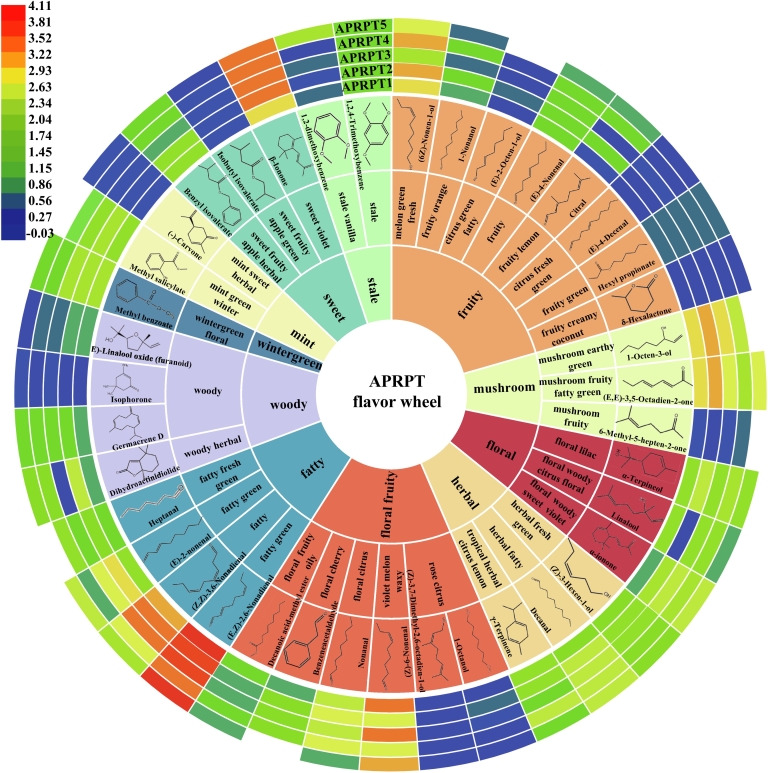
Flavor wheel showing the characteristic volatiles in ancient plant ripened pu-erh tea. Note: Heatmaps represent odor activity values.

The aroma types of the volatile compounds with OAVs >1 were identified to highlight the characteristic aromas of the APRPTs. Based on these types, a flavor wheel was constructed to better represent the distinct aromas of the teas **(**Fig. 6**)**. The aroma odors of the APRPTs were primarily categorized as fruity, floral, floral fruity, sweet, mushroom, herbal, stale, woody, mint, wintergreen, and fatty were further classified into 11 subcategories **(**Fig. 6**)**. The first primary category included representative compounds with fruity aromas, such as (6Z)-nonen-1-ol, 1-nonanol, (E)-2-octen-1-ol, (E)-4-nonenal, citral, (E)-4-decenal, hexyl propionate, and δ-hexalactone, with average OAVs of 1697.94, 11.70, 2.81, 18.32, 14.78, 2.19, 6.15, and 2.34, respectively **(**Fig. 5, Fig. 6**)**. Among them, nonanol has also been found in other pu-erh tea samples and has an aroma described as orange and fruit (Wang, He, Zhang, Du and Xu, 2020). The second category included representative compounds such as α-terpineol, linalool, and α-ionone **(**Fig. 6**)**. Pu-erh tea has also been reported to contain α-terpineol and this gives tea a relatively strong odor intensity (Wang et al., 2022). Linalool in tea has been reported to be synthesized by linalool synthase through the catalysis of the geranyl pyrophosphate precursor (Guo et al., 2019) and α-ionone in tea is produced through enzymatic reactions or non-enzymatic degradation, with its primary precursor being α-carotene (Ho et al., 2015). Here, the average OAVs for α-terpineol, linalool, and α-ionone in the APRPTs were 167.45, 66.54, and 187.55, respectively, and the samples had a floral aroma. The third category included decanoic acid, methyl ester, benzeneacetaldehyde, nonanal, (Z)-6-nonenal, (Z)-3,7-dimethyl-2,6-octadien-1-ol, and 1-octanol **(**Fig. 6**)**. Benylacetaldehyde has been reported to be the primary volatile compound in teas with floral aroma properties (Wilfried et al., 2008) and has been frequently identified in pu-erh tea. Nonanal, with its fruity and fatty aroma, has been shown to make a key contribution to the formation of pu-erh tea aroma (Pang et al., 2019). These compounds led to floral fruity aromas and had average OAVs of 13.71, 27.03, 511.30, 5503.05, 1.03, and 2.66 in the APRPT samples, respectively Fig. 5.

β-ionone (average OAV = 3401.67), isobutyl isovalerate (average OAV = 1.72), and benzyl isovalerate (average OAV = 37.83) are representative compounds in category 4 **(**Fig. 5, Fig. 6**)**. They are also abundant in other traditional dark teas, such as Fu-brick tea and Liu-pao tea (Ma et al., 2023; Pang et al., 2019). These volatile compounds lead to a sweet aroma and play a coordinating role to the overall aroma. Representative compounds in category 5 include 1-octen-3-ol (average OAV = 1552.65), (E,*E*)-3,5-octadien-2-one (average OAV = 1096.37), and 6-methyl-5-hepten-2-one (average OAV = 2.11) **(**Fig. 5, Fig. 6**)**. These compounds lead to a mushroom aroma and have been repeatedly identified as characteristic volatile compounds in pu-erh tea (Wang et al., 2022). Among them, the OAVs for 1-octen-3-ol were 16.74–82.56 higher in this study than those reported by Tian et al. (2024). The high values in this study may have caused the strong mushroom aroma, which was a characteristic of the APRPT samples used in this study. Typical compounds in category 6 (herbal) included (Z)-3-hexen-1-ol, decanal, and γ-terpinene **(**Fig. 6**)**. Decanal, with an average OAV of 348.22 **(**Fig. 5**)**, is considered to be a significant volatile compound because of its strong odor presence in pu-erh tea (Lv et al., 2012). Category 7 included 1,2,4-trimethoxybenzene and 1,2-dimethoxybenzene, with average OAVs of 19.65 and 27.63, respectively. They are associated with stale aromas **(**Fig. 5, Fig. 6**)**. Methoxybenzene is recognized as a key volatile compound in pu-erh tea and significantly contributes to a stale aroma (Wang et al., 2022). Additionally, methoxybenzenes have been consistently identified as characteristic volatile compounds in other types of dark teas (Liu et al., 2022). Here the 1,2,4-trimethoxybenzene and 1,2-dimethoxybenzene contents were 29.75–370.42 and 44.88–456.13 lower than those reported by (Tian et al., 2024), respectively. These low values may have reduced the stale flavor levels and meant that it was not a characteristic aroma of the APRPTs used in this study.

Category 8 compounds included (E)-linalool oxide (furanoid), isophorone, germacrene D, and dihydroactinidiolide. **(**Fig. 6**)**. Dihydroactinidiolide has been reported to be produced by microbial activity, oxidative degradation, or the thermal degradation of *β*-carotene during the fermentation of pu-erh tea (Lv et al., 2015) and isophorone is found in four types of dark tea (Ma et al., 2020). (E)-linalool oxide (furanoid), isophorone, germacrene D, and dihydroactinidiolide had average OAVs of 7.04, 2.12, 19.77, and 357.77, respectively. They are associated with a woody aroma and coordinate the overall aroma of APRPT. Typical compounds in category 9 (mint) included methyl salicylate and (−)-carvone **(**Fig. 6**)**. Methyl salicylate, a characteristic volatile compound, is synthesized from salicylic acid by salicylic acid carboxyl methyltransferase and is present in several types of tea. It contributes to a mint aroma (Deng et al., 2017; Ma et al., 2020). Carvone provides mint, fennel, and basil odors and has been identified as a characteristic volatile compound in pu-erh tea (Lv et al., 2012). The average OAVs for methyl salicylate and (−)-carvone in the APRPT samples were 100.95 and 1.39, respectively, which meant that they contributed to the overall aroma. Methyl benzoate was in category 10, it produces a wintergreen type aroma, and had an average OAV of 146.16 **(**Fig. 5, Fig. 6**)**. Category 11 (fatty) included aldehydes such as heptanal, (E)-2-nonenal, (Z,Z)-3,6-nonadienal, and (E,Z)-2,6-nonadienal. They produce fatty aromas and had average OAVs of 27.68, 827.02, 3173.12, and 13,195.07, respectively **(**Fig. 5, Fig. 6**)**. Studies have shown that heptanal in tea is produced by the thermal degradation of palmitoleic and oleic acids (Ho et al., 2015), (E)-2-nonenal has been identified as having metallic, fatty, and leafy odors, has been found in pu-erh tea, and has a high flavor dilution factor (Pang et al., 2019). (E, Z)-2,6-nonadienal is considered to be an extremely potent volatile compound in pu-erh tea and is produced by the oxidation of fatty acids (Ho et al., 2015). These compounds are also essential aroma components of APRPT and help coordinate the overall aroma.

In summary, 39 volatile compounds had OAVs greater than one and were associated with fruity, floral, floral fruity, sweet, mushroom, herbal, stale, woody, mint, wintergreen, and fatty aromas. These compounds are responsible for the special aroma associated with APRPT or play a coordinating role in overall aroma. Among them, 1-octen-3-ol, (E,E)-3,5-octadien-2-one, and 6-methyl-5-hepten-2-one may cause the long-lasting mushroom aroma associated with APRPT. The flavor wheel created in this study showing the aromatic properties of compounds with varying molecular structures can serve as a foundational guide for establishing a quantitative evaluation standard for the aroma quality of APRPT.

## 4 Conclusions

The characteristic flavor and chemical basis of five representative a APRPTs were investigated. They all had a reddish and thick infusion color, a mellow taste, and a characteristic mushroom aroma. Theabrownins, soluble sugar, caffeine, and gallic acid were identified as being responsible for the reddish infusion color and mellow taste. A total of 157 volatile compounds from eight classes of chemicals were identified in the APRPTs, among them, the OAVs of 39 compounds were greater than one and these were identified as characteristic volatile compounds in APRPTs. A flavor wheel was constructed based on 11 aroma types and three compounds were identified as being responsible for the mushroom aroma, which were 1-octen-3-ol (average OAV = 1552.65), (E,E)-3,5-octadien-2-one (average OAV = 1096.37), and 6-methyl-5-hepten-2-one (average OAV = 2.11). Together, these results can be used to further investigate the quality characteristics and formation mechanisms associated with APRPTs. Future research should conduct more aroma recombination experiments and omission tests to clearly verify the contribution of characteristic flavor.

## Ethical statement and sensory consent

Ethical permission, to conduct a human sensory study, was granted the Yunnan Agricultural University Institutional Review Board Committee. The panelists involved in the sensory evaluation provided informed consent by affirming the following statement before participating in the survey: “I have carefully read the information and fully understand my rights as a potential subject in a research experiment. I am aware that my responses are confidential, and I agree to participate in this study.” They had the option to withdraw from the survey at any time without providing a reason. The applied tea samples were safe for consumption.

## CRediT authorship contribution statement

**Teng Wang:** Writing – original draft, Methodology, Investigation, Formal analysis, Data curation. **Nianguo Bo:** Software, Investigation. **Yiqing Guan:** Software, Investigation. **Dihan Yang:** Software, Investigation. **Qiuyue Chen:** Software, Investigation. **Yanhui Guan:** Software, Investigation. **Songzhi Liu:** Resources, Investigation. **Zhihui Wang:** Resources, Investigation. **Hongxing Duan:** Investigation. **Yan Ma:** Writing – review & editing, Supervision, Project administration, Funding acquisition. **Ming Zhao:** Writing – review & editing, Supervision, Project administration, Funding acquisition.

## Declaration of competing interest

We declare that all the authors have no conflicts of interest in this research and we have seen and approved the final version submitted.

## Data Availability

Data will be made available on request.

## Appendix A Supplementary data

Table S1 Image 1

Table S2 Image 2
