# Supplementary material for: An integrated flavoromics and chemometric analysis of the characteristic flavor, chemical basis and flavor wheel of ancient plant ripened pu-erh tea
Source: Food Chem X. 2025 Feb 15;26:102278. doi: 10.1016/j.fochx.2025.102278 (PMC11880732; doi:10.1016/j.fochx.2025.102278)
Supplement: Supplementary file 1 — Table S1 [file mmc1.docx]

**Table S1** Dose-over-threshold (DOT) analysis of taste compounds in ancient plant ripened pu-erh tea.

| Taste attributes ^a^ | Compounds | Thresholds (μg/L)^b^ | DOT^c^ | | | | |
| --- | --- | --- | --- | --- | --- | --- | --- |
|  |  |  | ATRPT1 | ATRPT2 | ATRPT3 | ATRPT4 | ATRPT5 |
| Bitterness | Thearubigins | 140 | 0.33 | 0.31 | 0.26 | 0.28 | 0.34 |
|  | Catechin | 290 | 0.03 | 0.02 | 0.06 | 0.07 | 0.02 |
|  | Epicatechin | 600 | 0.01 | 0.02 | 0.04 | 0.03 | 0.02 |
|  | Epigallocatechin | 350 | 0.03 | 0.03 | 0.05 | 0.11 | 0.05 |
|  | Gallocatechin | 1630 | 0.04 | 0.04 | 0.03 | 0.04 | 0.03 |
|  | Catechin gallate | 170 | 0.03 | 0 | 0.02 | 0.02 | 0.01 |
|  | Epicatechin-3-gallate | 200 | 0.08 | 0.07 | 0.05 | 0.04 | 0.05 |
|  | Epigallocahechin-3-gallate | 300 | 0.07 | 0.02 | 0.04 | 0.02 | 0.02 |
|  | Gallocatechin gallate | 180 | 0.06 | 0 | 0.07 | 0.06 | 0.04 |
|  | Caffeine | 97.1 | 4.86 | 4.21 | 5.38 | 4.14 | 5.21 |
|  | Gallic acid | 140 | 0.71 | 0.62 | 0.87 | 0.21 | 0.58 |
|  | Quercetin | 20 | 0.07 | 0 | 0.05 | 0.05 | 0.03 |
|  | Myricetin | 10 | 0.23 | 0.18 | 0.24 | 0.18 | 0.12 |
|  | Kaempferol | 50 | 0.01 | 0.01 | 0.01 | 0.01 | 0.01 |
|  | Histidine | 7447 | 0 | 0 | 0 | 0 | 0 |
|  | Arginine | 4355 | 0 | 0 | 0 | 0 | 0 |
|  | Phenylalanine | 2650 | 0 | 0 | 0 | 0 | 0 |
|  | Proline | 2330 | 0 | 0 | 0 | 0 | 0 |
|  | Valine | 1950 | 0 | 0 | 0 | 0 | 0 |
|  | Leucine | 1450 | 0 | 0 | 0 | 0 | 0 |
|  | Isoleucine | 1190 | 0 | 0 | 0 | 0 | 0 |
|  | Tyrosine | 1090 | 0 | 0 | 0 | 0 | 0 |
| Astringence | Thearubigins | 800 | 0.06 | 0.05 | 0.04 | 0.05 | 0.06 |
|  | Catechin | 119.01 | 0.06 | 0.04 | 0.15 | 0.16 | 0.05 |
|  | Epicatechin | 269.95 | 0.03 | 0.04 | 0.09 | 0.07 | 0.04 |
|  | Epigallocatechin | 159.26 | 0.06 | 0.07 | 0.11 | 0.25 | 0.1 |
|  | Gallocatechin | 165.39 | 0.39 | 0.44 | 0.33 | 0.43 | 0.33 |
|  | Catechin gallate | 110.59 | 0.04 | 0 | 0.03 | 0.03 | 0.02 |
|  | Epicatechin-3-gallate | 115.02 | 0.14 | 0.12 | 0.09 | 0.06 | 0.09 |
|  | Epigallocahechin-3-gallate | 87.09 | 0.25 | 0.08 | 0.13 | 0.06 | 0.08 |
|  | Gallocatechin gallate | 178.79 | 0.06 | 0 | 0.07 | 0.06 | 0.04 |
|  | Gallic acid | 34.024 | 2.93 | 2.53 | 3.57 | 0.88 | 2.37 |
|  | Theanine | 1050 | 0 | 0 | 0 | 0 | 0 |
| Sweetness | Threonine | 4764.8 | 0 | 0 | 0 | 0 | 0 |
|  | Serine | 3152.7 | 0 | 0 | 0 | 0 | 0 |
|  | Proline | 2993.38 | 0 | 0 | 0 | 0 | 0 |
|  | Valine | 2230 | 0 | 0 | 0 | 0 | 0 |
|  | Methionine | 750 | 0 | 0 | 0 | 0 | 0 |
|  | Alanine | 712.744 | 0 | 0 | 0 | 0 | 0 |
|  | Glycine | 2252.01 | 0 | 0 | 0 | 0 | 0 |
|  | Cysteine | 242 | 0.02 | 0.02 | 0.02 | 0.02 | 0.02 |
| Sourness | Glutamic acid | 147 | 0.03 | 0.02 | 0.02 | 0.02 | 0.02 |
|  | gamma-aminobutyric acid | 40 | 0.03 | 0.05 | 0.05 | 0.06 | 0.06 |
|  | Aspartate | 143 | 0.03 | 0.03 | 0.02 | 0.03 | 0.02 |
|  | Gallic acid | 187 | 0.53 | 0.46 | 0.65 | 0.16 | 0.43 |
| Umami | Glutamic acid | 29.426 | 0.14 | 0.12 | 0.09 | 0.12 | 0.09 |
|  | Theanine | 3532.752 | 0 | 0 | 0 | 0 | 0 |
|  | Aspartate | 532.412 | 0.01 | 0.01 | 0.01 | 0.01 | 0.01 |

Note: ^a,b^ The taste attributes and thresholds were sourced from Susanne and Thomas (2005) and [Van Gemert (2011)](https://www.ncbi.nlm.nih.gov/pmc/articles/PMC9478030/#B28), The DOT value of each taste compound is calculated by dividing its concentration by its odor threshold (Susanne & Thomas, 2005).
