# Supplementary material for: An integrated flavoromics and chemometric analysis of the characteristic flavor, chemical basis and flavor wheel of ancient plant ripened pu-erh tea
Source: Food Chem X. 2025 Feb 15;26:102278. doi: 10.1016/j.fochx.2025.102278 (PMC11880732; doi:10.1016/j.fochx.2025.102278)
Supplement: Supplementary file 2 — Table S2 [file mmc2.docx]

**Table S2.** Concentration of volatiles in ancient plant ripened pu-erh tea.

| Types of compounds | Compounds | CAS | Retention index (RI) | | Concentration (μg/kg dry weight of tea leaves)^c,d^ | | | | |
| --- | --- | --- | --- | --- | --- | --- | --- | --- | --- |
|  |  |  | RI (DB)^a^ | RI (NIST)^b^ | APRPT1 | APRPT2 | APRPT3 | APRPT4 | APRPT5 |
| Hydrocarbons  （35） | Cyclosativene | 22469-52-9 | 1388.34 | 1368 | 2096.05±438.44^a^ | 1381.71±105.11^b^ | 360.28±5.32^c^ | 482.13±17.92^c^ | 402.77±31.24^c^ |
|  | 1-Undecyne | 2243-98-3 | 1095.38 | 1095 | 213.84±26.01^ab^ | 246.07±24.27^a^ | 218±11.73^ab^ | 256.47±11.56^a^ | 180.6±43.35^b^ |
|  | 1,4-Dimethyl-2,5-bis(1-methylethyl)benzene | 10375-96-9 | 1413.24 | 1403 | 164.44±13.86^a^ | 168.17±0.72^a^ | 92.32±15.62^c^ | 110.94±4.53^b^ | 89.16±6.14^c^ |
|  | α-Bisabolene | 17627-44-0 | 1509.89 | 1518 | 227.53±22.34a | 241.85±13.95a | 78.37±2.85c | 92.87±26.09^c^ | 127.74±4.41^b^ |
|  | (1-Methyl-1-propylpentyl)benzene | 54932-91-1 | 1510.30 | 1504 | 87.93±13.56^a^ | 87.8±18.28^a^ | 36.9±2^b^ | 51.48±3.6^b^ | 44.07±0.53^b^ |
|  | 9-Methyl-1-undecene | 74630-41-4 | 1143.95 | 1152 | 30.97±1.87^a^ | 33.24±2.13^a^ | 30.23±2.58^b^ | 20.85±9.02^b^ | 31.00±3.72^a^ |
|  | Sativene | 3650-28-0 | 1388.19 | 1396 | 360.37±21.51^a^ | 230.81±3.76^b^ | 64.63±1.37^c^ | 86.86±3.71^b^ | 74.72±4.92^b^ |
|  | 3,6-Dimethylundecane | 17301-28-9 | 1209.75 | 1210 | 157.76±53.68^a^ | 210.29±31.08^a^ | 182.61±3.37^a^ | 210.87±9.21^a^ | 164.36±15.57^a^ |
|  | 5,7-Dimethylundecane | 17312-83-3 | 1195.32 | 1190 | 382.35±33.69^ab^ | 392.6±10.01^ab^ | 319.66±2.83^c^ | 355.01±35.5^bc^ | 411.59±12.42^a^ |
|  | 5-(2-Methylpropyl)nonane | 62185-53-9 | 1194.36 | 1185 | 70.46±9.15^a^ | 68.66±6.27^a^ | 61.47±3.08^ab^ | 70.24±1.97^a^ | 51.86±0.61^b^ |
|  | β-himachalene | 1461-03-6 | 1509.89 | 1500 | 81.95±11.17^a^ | 71.84±6.75^a^ | 24.61±1.90^c^ | 37.96±3.09^b^ | 34.52±2.03^bc^ |
|  | 3,8-Dimethylundecane | 17301-30-3 | 1195.32 | 1185 | 537.5±52.04^bc^ | 627.84±7.6^a^ | 446.4±9.86^c^ | 518.03±12.48^d^ | 581.55±19.94^b^ |
|  | 2-Methylundecane | 7045-71-8 | 1171.80 | 1164 | 82.84±8.25^c^ | 589.03±36.91^a^ | 78.56±13.73^cd^ | 486.09±17.53^b^ | 46.31±8.99^d^ |
|  | 5-Methyl-5-propylnonane | 17312-75-3 | 1217.51 | 1229 | 62.39±14.27^a^ | 77.49±11.33^a^ | 68.35±4.74^a^ | 77.01±4.02^a^ | 60.78±7.8^a^ |
|  | Ethylbenzene | 100-41-4 | 864.37 | 863 | 48.1±4.49^b^ | 88.19±15.19^a^ | 39.36±3.46^bc^ | 92.26±7.17^a^ | 29.3±2.09^c^ |
|  | 2,6,10-Trimethyltetradecane | 14905-56-7 | 1536.39 | 1539 | 56.05±17.44^a^ | 60.59±7.76^a^ | 47.69±3.31^a^ | 60.28±4.41^a^ | ND |
|  | 3-Ethyl-3-methyldecane | 17312-66-2 | 1240.00 | 1229 | 62.2±12.02^b^ | 78.26±10.92^a^ | 66.32±1.17^ab^ | 73.62±5.8^ab^ | 61.06±0.97^b^ |
|  | 4,4-Dimethylundecane | 17312-68-4 | 1217.56 | 1229 | 42.54±10.92^a^ | 50.74±8.25^a^ | 44.99±1.6^a^ | 54.68±3.83^a^ | 41.84±5.63^a^ |
|  | 4-Methyltetradecane | 25117-24-2 | 1457.40 | 1459 | 258.53±38.43^b^ | 328.48±27.25^a^ | 202.53±2.44^cd^ | 235.14±14.09^bc^ | 164.65±5.2^d^ |
|  | α-Cedrene | 469-61-4 | 1388.31 | 1411 | 1541.44±95.99^a^ | 916.42±18.9^b^ | 302.7±72.07^c^ | 347.01±16.87^c^ | 349.84±121.75^c^ |
|  | α-Funebrene | 50894-66-1 | 1388.19 | 1400 | 1824.22±55.75^a^ | 1180.07±17.55^b^ | 326.7±4.97^d^ | 437.65±20.68^c^ | 373.01±28.67^d^ |
|  | 4,6-Dimethyl-dodecane, | 61141-72-8 | 1322.12 | 1325 | 173.37±38.01^ab^ | 195.32±24.1^ab^ | 159.22±9.16^b^ | 181.05±6.08^ab^ | 214.15±5.65^a^ |
|  | 3-Ethyl-2-methyl-1,3-hexadien | 61142-36-7 | 1025.74 | 1031 | 109.52±14.15^a^ | 124.69±11.23^a^ | 66.15±6.56^b^ | 112.74±12.55^a^ | 66.60±7.64^b^ |
|  | 1,10-Undecadiene | 13688-67-0 | 1095.88 | 1095 | 284.88±14.93^b^ | 408.61±40.78^a^ | 273.15±6.19^b^ | 401.3±17.62^a^ | 184.88±6.96^c^ |
|  | (E)-2-Tetradecene | 35953-54-9 | 1394.98 | 1406 | 159.42±20.44^b^ | 187.74±10^a^ | 114.88±10.65^c^ | 115.38±6.9^c^ | 45.86±3.22^d^ |
|  | Hexadecane | 544-76-3 | 1594.46 | 1600 | 211.23±27.9^c^ | 297.35±26.32^b^ | 181.72±38.72^cd^ | 141.87±1.08^d^ | 793.42±41.04^a^ |
|  | γ-Cadinene | 39029-41-9 | 1510.04 | 1513 | 75.32±7.28^a^ | 83.06±17.20^a^ | 34.63±9.28^b^ | 44.78±6.45^b^ | 37.79±1.07^b^ |
|  | Germacrene D | 23986-74-5 | 1485.67 | 1481 | 15.34±7.51^b^ | 21.12±2.61^ab^ | 26.64±6.65^a^ | 26.93±1.48^a^ | 28.58±1.36^a^ |
|  | Pentadecane | 629-62-9 | 1494.35 | 1499 | 773.45±173.61^b^ | 1084.51±79.04^a^ | 689.69±56.67^b^ | 755.49±42.76^b^ | 450.81±96.13^c^ |
|  | γ-Elemene | 29873-99-2 | 1428.40 | 1433 | 107.49±6.5^c^ | 214.46±17.36^a^ | 154.06±3.24^b^ | 117.22±27.15^c^ | 216.97±1.03^a^ |
|  | Tetradecane | 629-59-4 | 1395.00 | 1400 | 978.9±88.44^b^ | 1176.05±52.21^a^ | 774.01±35.77^c^ | 679.29±17.38^d^ | 267.67±6.32^e^ |
|  | cis-*β*-farnesene | 28973-97-9 | 1437.85 | 1444 | 29.73±2.7^d^ | 81.32±6.69^a^ | 40.35±0.89^bc^ | 48.37±6.89^b^ | 38.23±3.71^c^ |
|  | γ-Terpinene | 99-85-4 | 1070.47 | 1060 | 191.88±49.71^a^ | 146.22±12.42^b^ | 52.12±3.04^d^ | 101.51±8.67^c^ | 39.91±3.17^d^ |
|  | Decane | 124-18-5 | 996.00 | 1000 | 185.58±16.98^ab^ | 204.78±6.71^a^ | 141±16.74^c^ | 171.55±5.54^b^ | 199.19±12.71^a^ |
|  | Toluene | 108-88-3 | 750.89 | 763 | 138.57±12.6^c^ | 148.59±4.83^bc^ | 160.01±5.97^b^ | 177.17±11.28^a^ | 98±3.6^d^ |
| Alcohols  （34） | 1-Cyclohexene-1-propanol | 3293-47-8 | 1439.29 | 1452 | 47.3±6.74^a^ | 42.72±8.79^a^ | 22.22±2.2^bc^ | 28.57±2.06^b^ | 16.6±0.64^c^ |
|  | Lilac Alcohol C | 33081-36-6 | 1212.31 | 1219 | 97.65±19.02^a^ | 81.78±8.75^ac^ | 60.65±6.28^c^ | 68.23±5.49^bc^ | 58.48±1.99^c^ |
|  | 6-Undecanol | 23708-56-7 | 1276.23 | 1277 | 25.76±6.95^a^ | 30.96±3.79^a^ | 26.9±0.75^a^ | 32.01±0.48^a^ | 33.6±5.3^a^ |
|  | 2-Butyl-2-Octenal | 13019-16-4 | 1386.86 | 1378 | 167.2±5.17^a^ | 118.1±2.64^b^ | 53.69±13.96^d^ | 53.65±1.09^d^ | 74.66±4.01^c^ |
|  | Cyclooctanemethanol | 3637-63-6 | 1070.40 | 1073 | 219.83±12.32^b^ | 318.24±105.58^a^ | 125.38±4.95^c^ | 218.14±13.86^b^ | 82.57±23.14^c^ |
|  | (E)-3-Nonen-1-ol | 10339-61-4 | 1144.62 | 1143 | 76.14±5.25^a^ | 40.46±4.80^c^ | 38.06±6.51^c^ | 19.02±1.28^d^ | 49.34±2.27^b^ |
|  | (E)-2-Decen-1-ol | 18409-18-2 | 1252.57 | 1257 | 658.24±46.97^b^ | 737.7±24.48^a^ | 427.84±10.08^c^ | 684.49±34.66^ab^ | 701.6±77.98^ab^ |
|  | (6Z)-Nonen-1-ol | 35854-86-5 | 1172.07 | 1171 | 1329.56±120.41^b^ | 2615.97±547.05^a^ | 1240.99±38.9^b^ | 2554.02±146.48^a^ | 749.15±9.35^c^ |
|  | (E)-2-Nonen-1-ol | 31502-14-4 | 1172.71 | 1168 | 104.33±13.73^cd^ | 398.53±51.04^a^ | 120.63±20.96^c^ | 245.17±18.23^b^ | 72.67±9.59^d^ |
|  | (Z)-3-Nonen-1-ol | 10340-23-5 | 1144.74 | 1154 | 86.91±11.04^a^ | ND | 42.71±3.74^b^ | ND | 43.18±1.26^b^ |
|  | (E)-2-Undecen-1-ol | 75039-84-8 | 1366.03 | 1365 | 157.98±14.75^b^ | 193.38±4.54^a^ | 120.15±2.47^c^ | 155.17±3.92^b^ | 115.98±0.57^c^ |
|  | Isopinocarveol | 6712-79-4 | 1171.61 | 1178 | 173.12±16.89^b^ | 347.63±101.47^a^ | 172.49±13.95^b^ | 314.86±16.79^a^ | 123.57±2.69^b^ |
|  | 2-Nonen-1-ol | 22104-79-6 | 1105.70 | 1105 | 297.59±28.27^b^ | 377.7±34.05^a^ | 189.59±3.10^c^ | 159.07±12.63^c^ | 100.47±8.33^d^ |
|  | 2,4-Decadien-1-ol | 14507-02-9 | 1275.84 | 1274 | 29.22±3.13^c^ | 34.62±4.76^ab^ | 26.5±0.18^c^ | 38.34±0.96^a^ | 29.66±2.25^bc^ |
|  | Coniferyl alcohol | 458-35-5 | 1484.86 | 1485 | 1595.1±563.25^a^ | 1547.03±101.72^a^ | 1538.64±144.13^a^ | 1712.38±64.07^a^ | 1552.36±185.87^a^ |
|  | α-Terpineol | 10482-56-1 | 1190.86 | 1195 | 244.04±8.61^c^ | 1697.17±170.99^a^ | 348.01±75.12^c^ | 873.24±144.82^b^ | 253.6±4.85^c^ |
|  | 4-Methyl-benzenemethanol | 589-18-4 | 1096.35 | 1106 | 116.5±16.56^b^ | 142.07±15.29^a^ | 90.34±4.42^c^ | 137.5±4.79^a^ | 44.17±1.07^d^ |
|  | DL-1-Phenethylalcohol | 98-85-1 | 1065.62 | 1061 | 109.15±30.68^a^ | 68.44±4.15^b^ | 33.17±1.84^bc^ | 55.35±3.49^ab^ | 12.08±10.51^c^ |
|  | Caryophyllenolexcloveleafoil | 4586-22-5 | 1566.38 | 1566 | 50.61±4.45^a^ | 28.31±2.24^c^ | 31.4±1.39^c^ | 26.97±1.55^c^ | 48.43±9.38^b^ |
|  | 1-Dodecanol | 112-53-8 | 1485.24 | 1474 | 73.94±21.03^c^ | 95.35±6.84^ab^ | 105.76±3.36^a^ | 98.1±8.60^a^ | 111.91±13.36^a^ |
|  | (E,Z)-3,6-Nonadien-1-ol | 56805-23-3 | 1144.33 | 1156 | 131.26±27.3^a^ | ND | 43.56±3.26^c^ | ND | 74.33±1.16^b^ |
|  | (Z)-3,7-Dimethyl-2,6-octadien-1-ol | 106-25-2 | 1241.80 | 1230 | 49.24±4.60^a^ | 45.88±3.74^a^ | 55.64±9.18^a^ | 50.88±3.76^a^ | 50.58±3.21^a^ |
|  | Levomenthol | 2216-51-5 | 1171.63 | 1177 | 39.11±12.88^c^ | 133.92±12.22^a^ | 37.32±9.39^c^ | 84.84±3.87^b^ | 27.03±0.62^c^ |
|  | (E,Z)-2,6-Nonadienol | 28069-72-9 | 1172.18 | 1170 | 615.7±57.04^b^ | 1125.25±252.86^a^ | 469.05±144.73^c^ | 1143.59±79.22^a^ | 339.56±9.71^c^ |
|  | 1-Nonanol | 143-08-8 | 1172.50 | 1171 | 42.05±6.48^c^ | 124.71±10.4^a^ | 31.6±4.97^c^ | 83.82±15.1^b^ | 27.99±3.11^c^ |
|  | (E)-2-Octen-1-ol | 18409-17-1 | 1070.34 | 1068 | 67.26±9.52^b^ | 109.18±38.69^a^ | 39.27±1.29^b^ | 65.18±2.24^b^ | ND |
|  | Linalool | 78-70-6 | 1096.45 | 1101 | 396.82±40.64^b^ | 600.53±60.82^a^ | 356.08±11.57^b^ | 561.92±28.69^a^ | 80.93±1.2^c^ |
|  | 1-Octanol | 111-87-5 | 1070.16 | 1070 | 72.6±35.19^ab^ | 92.35±27.09^a^ | 26.36±4.86^b^ | 53.01±3.08^ab^ | 47.96±29.88^ab^ |
|  | 1-Octen-3-ol | 3391-86-4 | 987.14 | 980 | 1144.3±172.32^c^ | 1991.9±224.93^b^ | 1186.43±35.60^c^ | 3440.64±167.69^a^ | ND |
|  | (Z)-3-Hexen-1-ol | 928-96-1 | 857.77 | 856 | 329.14±27.03^ab^ | 366.12±37.49^a^ | 228.69±130.8^bc^ | 150.6±13.51^c^ | 371.56±31.57^a^ |
|  | 5-Undecanol | 37493-70-2 | 1296.23 | 1288 | 82.44±4.57^a^ | 56.54±12.17^b^ | 50.98±15.66^bc^ | 33.88±14.^23cd^ | 27.15±2.1^d^ |
|  | (S)-(-)-2-Methyl-6-methylene-7-octen-4-ol | 35628-05-8 | 1105.15 | 1097 | 3107.36±426.83^a^ | 3153.63±273.22^a^ | 1209.48±55.69^b^ | 1270.13±155.71^b^ | 577.82±15.35^c^ |
|  | Isopinocampheol | 27779-29-9 | 1171.37 | 1179 | 229.83±25.2^c^ | 537.78±31.36^a^ | 266.21±18.15^c^ | 399.3±9.42^b^ | 146.72±34.33^d^ |
|  | 4-Phenyl-2-butanol | 2344-70-9 | 1252.61 | 1262 | 125.81±6.66^ab^ | 145.21±3.74^a^ | 78.95±17.44^c^ | 138.02±5.07^ab^ | 107.47±36.6^bc^ |
| Esters  （29） | Isobutyl isovalerate | 589-59-3 | 996.10 | 1005 | 52.07±9.74^c^ | 60.3±14.15^ab^ | 46.41±3.64^c^ | 61.17±6.04^ab^ | 72.09±7.28^a^ |
|  | 2-Methylbutyl caproate | 2601-13-0 | 1242.92 | 1247 | 33.78±40.33^c^ | 101.99±21.16^a^ | 87.98±9.71^ab^ | 75.51±3.5^abc^ | 58.72±11.72^a^ |
|  | Hexanoic acid cyclopentyl ester | 5413-59-2 | 1322.12 | 1325 | 28.19±7.02^a^ | 32.19±4.04^a^ | 27.01±3.87^a^ | 34.26±2.88^a^ | 35.15±1.55^a^ |
|  | (Z)-Pent-2-enyl butyrate | 42125-13-3 | 1096.45 | 1091 | 1177.07±105.23^b^ | 1729.92±177.41^a^ | 1104.74±45.14^b^ | 1653.05±88.34^a^ | 842.19±7.46^c^ |
|  | 5-Hexyl butyrate | 108058-75-9 | 1190.80 | 1183 | 62.41±3.78^c^ | 124.49±16.66^a^ | 44.66±4.26^d^ | 104.56±4.65^b^ | 49.66±3.81^cd^ |
|  | 3-Methylheptyl acetate | 72218-58-7 | 1095.93 | 1118 | 172.4±19.69^c^ | 219.02±22.96^ab^ | 176.92±10.38^c^ | 234.81±12.58^a^ | 197.73±6.08^bc^ |
|  | δ-Hexalactone | 823-22-3 | 1095.76 | 1095 | 57.86±8.33^b^ | 61.47±9.36^b^ | 57.43±3.16^b^ | 81.46±5.7^a^ | 56.08±3.06^b^ |
|  | Dihydroactinidiolide | 17092-92-1 | 1535.68 | 1532 | 232.71±29.41^c^ | 559.41±53.22^b^ | 630.99±12.28^a^ | 646.05±41.64^a^ | 637.43±19.73^a^ |
|  | Butyric acid (Z)-4-hexenyl ester | 69727-41-9 | 1190.05 | 1203 | 118.25±8.31^d^ | 524.79±44.44^a^ | 174.01±6.51^c^ | 354.61±22.36^b^ | 115.19±3.41^d^ |
|  | δ-Nonalactone | 3301-94-8 | 1395.00 | 1396 | 136.3±13.01^b^ | 163.38±7.00^a^ | 104.15±1.48^c^ | 98.7±3.42^c^ | 34.91±6.39^d^ |
|  | Methyl cinnamate | 1754-62-7 | 1388.19 | 1379 | 232.18±8.23^a^ | 157.21±17.96^b^ | 43.19±3.22^c^ | 55.79±3.10^c^ | 47.94±4.19^c^ |
|  | (E)-Butanoic acid | 106-29-6 | 1565.82 | 1562 | 32.21±6.6^bc^ | 35±2.8^b^ | 25.64±2.76^c^ | 31.95±3.26^bc^ | 87.51±4.2^a^ |
|  | Citronellyl isobutyrate | 97-89-2 | 1485.38 | 1483 | 75.71±16.62^b^ | ND | 123.6±10.76^a^ | 118.44±9.43^a^ | 128.78±10.36^a^ |
|  | Butyl caprate | 30673-36-0 | 1594.52 | 1590 | 31.71±4.61^c^ | 43.76±4.11^b^ | 30.26±3.23^c^ | 22.76±0.53^c^ | 180.03±11.87^a^ |
|  | (Z)-3,7-Dimethyl-2,6-octadien-1-ol acetate | 141-12-8 | 1371.02 | 1365 | 297.94±68.88^a^ | 117.81±9.24^b^ | 114.98±5.75^b^ | 154.33±16.79^b^ | 247.72±5.6^a^ |
|  | Decanoic acid-methyl ester | 110-42-9 | 1323.17 | 1326 | 75.18±11.26^a^ | 49.47±6.27^b^ | 54.64±4.7^b^ | 62.12±5.^12ab^ | 53.33±16.82^b^ |
|  | Nonyl acetate | 143-13-5 | 1296.20 | 1309 | 78.59±10.83^a^ | 59.69±0.89^b^ | 54.25±6.21^bc^ | 46.17±7.11^c^ | 47.92±1^c^ |
|  | (E)-Geranic acid methyl ester | 1189-09-9 | 1322.42 | 1322 | 142.53±18.02^b^ | 165.57±14.5^a^ | 68.76±6.65^c^ | 67.46±4.06^c^ | 56.67±2.58^c^ |
|  | Methyl salicylate | 119-36-8 | 1195.04 | 1200 | 2529.91±312.7^d^ | 5673.06±546.53^b^ | 3811.61±118.91^c^ | 8175.47±508.41^a^ | ND |
|  | Butyl hexanoate | 626-82-4 | 1195.32 | 1189 | 94.79±12.7^b^ | 106.78±4.27^a^ | 80.2±0.9^c^ | 89.53±0.31^bc^ | 97.62±3.04^ab^ |
|  | Hexyl butyrate | 2639-63-6 | 1195.02 | 1190 | 253.71±56.69^a^ | ND | 4.59±0.64^b^ | 2.99±0.21^b^ | ND |
|  | Methyl benzoate | 93-58-3 | 1096.76 | 1098 | 82.88±8.2^b^ | 111.1±11.6^a^ | 68.45±2.95^c^ | 105.42±3.61^a^ | 12.17±1.37^d^ |
|  | Furfuryl pentanoate | 36701-01-6 | 1241.75 | 1232 | 68.04±6.32^a^ | 69.28±8.94^a^ | 66.63±5^a^ | 73.93±4.1^a^ | 76.62±2.92^a^ |
|  | 2-Methyl butyl isovalerate | 2445-77-4 | 1095.93 | 1107 | 172.4±19.69^c^ | 219.02±22.96^ab^ | 176.92±10.38^c^ | 234.81±12.58^a^ | 197.73±6.08^bc^ |
|  | Hexyl propionate | 2445-76-3 | 1095.19 | 1108 | 55.28±4.25^a^ | 42.84±18.9^a^ | 46.26±5.4^a^ | 51.08±9.47^a^ | 50.54±0.94^a^ |
|  | Propyl 2-methylbutanoate | 2445-67-2 | 996.10 | 1004 | 52.07±9.74^c^ | 60.3±14.15^ab^ | 46.41±3.64^c^ | 61.17±6.04^ab^ | 72.09±7.28^a^ |
|  | 1-Isobutyryloxy-3,7-dimethyl-octa-2t,6-dien | 2345-24-6 | 1485.15 | 1475 | 51.26±9.04^b^ | 75.7±11.14^a^ | 75.78±0.55^a^ | 68.53±3.52^a^ | 77.07±3.1^a^ |
|  | (Z)-3-Hexen-1-ol propanoate | 33467-74-2 | 1105.22 | 1100 | 570.34±61.18^a^ | 616.48±51.07^a^ | 233.19±8.39^b^ | 257.25±15.06^b^ | 123.03±2.07^c^ |
|  | Benzyl isovalerate | 103-38-8 | 1388.19 | 1395 | 861±40.82^a^ | 553.24±10.79^b^ | 136.2±2.26^d^ | 183.28±6.52^c^ | 157.88±12.4^cd^ |
| Aldehydes  （21） | 7-Methyl-3-methylene-6-octenal | 55050-40-3 | 1144.83 | 1147 | 84.2±0.97^a^ | 52.97±3.39^b^ | 39.76±3.94^c^ | 29.41±0.7^d^ | 33.38±0.9^d^ |
|  | 2,6,6-Trimethylcyclohexa-1,4-dienecarbaldehyde | 162376-82-1 | 1096.96 | 1109 | 309.45±47.25^b^ | 464.34±62.01^a^ | 291.06±23.34^b^ | 411.13±23.83^a^ | 56.69±1.37^c^ |
|  | (E)-4-Decenal | 65405-70-1 | 1195.32 | 1198 | 54.15±6.24^b^ | 61.59±2.36^a^ | 44.62±2.14^c^ | 52.87±0.54^b^ | 60.64±1.16^a^ |
|  | 4-Methylbenzaldehyde | 104-87-0 | 1086.19 | 1084 | 97.03±14.16^ab^ | 106.25±10.74^a^ | 53.36±1.25^c^ | 83.71±4.92^b^ | 41.32±3.54^c^ |
|  | Lilac Aldehyde C | 53447-48-6 | 1171.47 | 1167 | 414.43±44.86^c^ | 939.17±89.14^a^ | 397.45±26.3^c^ | 752.05±37.63^b^ | 299.16±2.44^d^ |
|  | (Z)-6-Nonenal | 2277-19-2 | 1095.90 | 1104 | 738.26±67.44^b^ | 967.09±102.29^a^ | 743.96±43.09^b^ | 986.08±60.42^a^ | 416.74±15.59^c^ |
|  | (E)-4-Nonenal | 2277-16-9 | 1096.37 | 1105 | 40.97±6.35^b^ | 49.69±5.26^a^ | 40.52±5.99^b^ | 49.51±0.6^a^ | 20.82±0.96^c^ |
|  | Myrtenal | 564-94-3 | 1190.07 | 1193 | 32.94±3.86^c^ | 251.42±25.62^a^ | 56.94±3.43^c^ | 130.16±24.26^b^ | 35.31±0.05^c^ |
|  | 5-Hydroxymethylfurfural | 67-47-0 | 1241.78 | 1232 | 61.57±40.49^a^ | 69.62±6.53^a^ | 51.52±12.51^a^ | 66.31±4.7^a^ | 64.51±8.89^a^ |
|  | Dodecanal | 112-54-9 | 1394.97 | 1409 | 99.31±9.44^b^ | 128.66±6.29^a^ | 79.17±9.54^c^ | 80.59±3.44^c^ | 31.74±3.18^d^ |
|  | 4-(1-methylethyl)-Benzaldehyde | 122-03-2 | 1252.66 | 1246 | 29.6±1.74^b^ | 33.36±0.89^a^ | 20.09±1.2^c^ | 31.16±1.49^ab^ | 31.57±3.65^ab^ |
|  | Citral | 5392-40-5 | 1276.32 | 1273 | 54.11±16.48^b^ | 96.45±9.21^a^ | 100±0.69^a^ | 70.94±22.69^b^ | 47.95±2.2^b^ |
|  | Decanal | 112-31-2 | 1195.32 | 1206 | 30.8±6.38^bc^ | 37.74±5.5^ab^ | 26.98±1.53^c^ | 42.21±1.9^a^ | 36.38±6.86^abc^ |
|  | (E,Z)-2,6-Nonadienal | 557-48-2 | 1144.72 | 1155 | 113.55±11.04^a^ | 57.25±5.21^b^ | 39.29±2.74^c^ | 25.91±2.79^d^ | 60.88±3.59^b^ |
|  | (E)-2-nonenal | 18829-56-6 | 1153.39 | 1154 | 242.51±27.36^c^ | 546.76±37.05^a^ | 274.21±15.51^c^ | 440.16±20.65^b^ | 150.41±7.06^d^ |
|  | (Z,Z)-3,6-Nonadienal | 21944-83-2 | 1096.16 | 1100 | 134.83±6.34^d^ | 189.75±17.56^b^ | 166.17±9.43^c^ | 230.5±8.11^a^ | 72.03±4.05^e^ |
|  | Nonanal | 124-19-6 | 1095.95 | 1105 | 385.95±78.01^c^ | 532.82±55.78^b^ | 424.21±27.63^c^ | 513.11±20.43^b^ | 700.43±16.62^a^ |
|  | Benzeneacetaldehyde | 122-78-1 | 1047.47 | 1046 | 55.86±48.42^b^ | 157.29±19.92^a^ | 145.47±15.45^a^ | 181.91±13.18^a^ | ND |
|  | Heptanal | 111-71-7 | 898.01 | 901 | 94.35±11^d^ | 207.77±25.41^b^ | 135.38±21.81^c^ | 406.63±22.78^a^ | ND |
|  | 2-Isopropyl-5-methylhex-2-enal | 35158-25-9 | 1104.90 | 1106 | 119.76±12.92^a^ | 128.2±9.76^a^ | 45.1±2.09^b^ | 56.33±3.28^b^ | 24.81±0.68^c^ |
|  | (E,E)-2,4-Undecadienal | 30361-29-6 | 1439.67 | 1430 | 46.62±7.26^b^ | 62.3±5.51^a^ | 29.89±4.80^d^ | 38.46±1.84^c^ | ND |
| Ketones  （17） | Dehydrodihydroionone | 20483-36-7 | 1413.76 | 1424 | 260.64±18.84^b^ | 295.1±3.95^a^ | 175.6±12.54^d^ | 255.93±8.78^b^ | 201.75±5.85^c^ |
|  | 2,6,6-Trimethyl-2-cyclohexene-1,4-dione | 1125-21-9 | 1143.57 | 1147 | 140.61±15.26^bc^ | 221±24.51^a^ | 183.96±52.45^ab^ | 106.83±6.7^c^ | 113.45±1.54^c^ |
|  | 5-Methyl-2-(1-methylethylidene)cyclohexanone | 15932-80-6 | 1221.14 | 1212 | 41.03±12.26^c^ | 69.08±4.34^a^ | 64.42±2.62^a^ | 50.97±2.5^bc^ | 59.17±1.35^ab^ |
|  | 3-Methyl-2-cyclohexen-1-one | 1193-18-6 | 1065.71 | 1075 | 997.95±115.16^ab^ | 1056±120.44^a^ | 874.38±92.39^bc^ | 1076.71±68.92^a^ | 740.93±33.53^c^ |
|  | α-ionone | 127–41-3 | 1432.39 | 1429 | 41.23±10.5^c^ | 69.17±23.58^b^ | 94.39±10.52^a^ | 68.83±3.74^b^ | 101.47±2.35^a^ |
|  | (E)-2,6-Dimethylocta-5,7-dien-4-one | 6752-80-3 | 1144.29 | 1146 | 161.8±26.45^a^ | 64.44±8.44^bc^ | 57.22±4.04^c^ | 33.19±1.41^d^ | 86.72±11.16^b^ |
|  | 5-Methyl-4-Hexen-3-one | 13905-10-7 | 1086.22 | 1080 | 1394.03±139.4^b^ | 1565.9±141.66^a^ | 665.05±27.79^d^ | 1077±57.32^c^ | 454.84±8.66^e^ |
|  | (E,E)-3,5-Octadien-2-one | 30086-02-3 | 1086.31 | 1073 | 742.77±70.81^b^ | 826.75±77.11^a^ | 346.95±16.74^d^ | 575.41±31.32^c^ | 249.06±4.25^e^ |
|  | 4,6-Dimethyl-2,7-nonadien-5-one | 74630-80-1 | 1143.93 | 1138 | 37.77±3.14^ab^ | 42.96±4.33^a^ | 34.75±2.42^b^ | 32.57±3.60^b^ | 32.76±2.81^b^ |
|  | Isophorone | 78-59-1 | 1121.41 | 1123 | 459.88±58.01^b^ | 625±61.78^a^ | 356.66±53.35^cd^ | 385.34±27.58^bc^ | 291.39±7.28^d^ |
|  | β-Ionone | 14901-07-6 | 1487.26 | 1491 | 264.61±60.18^d^ | 839.76±89.46^b^ | 948.33±19.36^a^ | 684.51±57.38^c^ | 664.47±26.97^c^ |
|  | 6,10-Dimethyl-2-undecanone | 1604-34-8 | 1394.93 | 1408 | 45.8±6.47^b^ | 57.59±1.41^a^ | 32.59±3.51^c^ | 31.97±3.37^c^ | 12.27±0.54^d^ |
|  | D-Verbenone | 18309-32-5 | 1221.25 | 1228 | ND | 157.94±16.33^a^ | 120.88±4.88^b^ | 109.88±6.64^b^ | 83.96±0.35^c^ |
|  | Carvone | 99-49-0 | 1241.78 | 1242 | 47.67±5.39^ab^ | 50.16±6.83^ab^ | 46.88±0.83^ab^ | 50.96±3.15^a^ | 41.34±2^b^ |
|  | (-)-Carvone | 6485-40-1 | 1241.82 | 1250 | 109.01±8.40^b^ | 120.32±9.48^ab^ | 115.05±3.99^b^ | 116.62±8.14^ab^ | 130.92±4.93^a^ |
|  | 6-Methyl-5-hepten-2-one | 110-93-0 | 1326.06 | 1328 | 177.97±25.88^c^ | 255.3±31.70^b^ | 158.68±14.7^c^ | 461.6±24.42^a^ | ND |
|  | 8-Nonen-2-one | 5009-32-5 | 1086.05 | 1085 | 223.11±61.15^ab^ | 252.64±50.13^a^ | 96.4±5.06^c^ | 176.59±17.75^b^ | 49.52±1.04^c^ |
| Heterocycles  （6） | Hydrocoumarin | 119-84-6 | 1388.29 | 1387 | 444.27±17.78^a^ | 280.17±9.55^b^ | 66.03±1.77^d^ | 105.96±2.26^c^ | 89.13±6.82^d^ |
|  | (E)-Linalool oxide (furanoid) | 34995-77-2 | 1070.33 | 1075 | 2473.97±470.04^a^ | 1655.74±145.64^b^ | 719.39±35.58^d^ | 1243.96±71.55^c^ | 596.51±155.49^d^ |
|  | 1-(1H-pyrrol-2-yl)-ethanone | 1072-83-9 | 1069.59 | 1063 | 211.84±16.42^a^ | 200.62±10.25^ab^ | 58.34±41.65^c^ | 165±11.05^b^ | 79.05±0.94^c^ |
|  | 2-Hexyl-5-methyl-3-furanone | 33922-66-6 | 1436.71 | 1446 | 63.12±29.14^c^ | 160.47±21.96^a^ | 59.15±4.16^c^ | 102.62±8.21^b^ | 49.44±2.30^c^ |
|  | 2-Ethoxy-3-methylpyrazine | 32737-14-7 | 1065.66 | 1065 | 207.88±49.67^a^ | 139.04±8.90^b^ | 47.57±41.23^c^ | 119.2±12.60^b^ | 45.96±2.47^c^ |
|  | (2-Methylpropyl)pyrazine | 29460-92-2 | 1070.54 | 1074 | 1422.07±204.65^a^ | 1577.19±151.77^a^ | 681.48±32.35^c^ | 1199.51±62.36^b^ | 520.01±13.26^c^ |
| Acid  （4） | 2-Heptenoic acid | 18999-28-5 | 1086.12 | 1081 | 114.67±9.78^b^ | 128.57±12.11^a^ | 55.09±1.29^d^ | 90.59±7.08^c^ | 41.89±1.75^e^ |
|  | Heptanoic acid | 111-14-8 | 1086.12 | 1080 | 235.26±31.19^a^ | 251.61±22.36^a^ | 109.22±5.46^c^ | 176.13±10.36^b^ | 81.64±2.29^c^ |
|  | Undecylenic acid | 112-38-9 | 1456.62 | 1456 | 70.95±18.71^a^ | 78.1±4.26^a^ | 43.07±19.4^b^ | 61.7±2.73^ab^ | 57.27±6.81^ab^ |
|  | Octanoic acid | 124-07-2 | 1171.47 | 1172 | 69.98±11.74^b^ | 127.21±39.62^a^ | 58.18±4.69^b^ | 112.67±4.08^a^ | 36.18±30.89^b^ |
| Phenols  （2） | 2-Nitrophenol | 88-75-5 | 1144.55 | 1135 | 209.86±26.62^a^ | 103.65±13.35^b^ | ND | ND | ND |
|  | 2,4-Di-tert-butylphenol | 96-76-4 | 1508.88 | 1513 | 171.65±19.74^c^ | 198±28.46^ab^ | 165.54±12.72^c^ | 220.48±38.31^a^ | 92.63±6.21^bc^ |
| Methoxybenzenes  (4) | 1,2-Dimethoxybenzene | 91–16-7 | 1147.81 | 1148 | 12.8±1.86b | 5.8±3.81b | 14.35±7.72b | 8.13±1.24b | 396.89±35.31a |
|  | 1,2,4-Trimethoxybenzene | 135-77-3 | 1370.21 | 1374 | 1168.45±67.15a | 889.94±16.32b | 801.45±96.42b | 622.98±69.17c | 642.88±6.42c |
|  | 3,4-Dimethoxytoluene | 494-99-5 | 1239.67 | 1233 | 120.33±21.78b | 63.99±14.94c | 50.49±1.61c | 57.07±4.93c | 205.41±2.85a |
|  | (2-Methoxyethyl)-benzene | 3558-60-9 | 1095.42 | 1106 | 129.33±17.75a | 141.66±14.37a | 61.9±2.17c | 102±8.34b | 47.36±0.87c |
| Others  (5) | Camphor | 76-22-2 | 1144.60 | 1151 | 1101.96±136.6^a^ | 516.04±49.32^bc^ | 418.84±39.28^c^ | 218.67±10.91^d^ | 579.75±37.23^b^ |
|  | Perillen | 539-52-6 | 1095.92 | 1101 | 654.15±36.99^b^ | 900.06±90.4^a^ | 616.55±37.03^b^ | 893.35±45.19^a^ | 370.36±9.24^c^ |
|  | α-Pinene Oxide | 1686-14-2 | 1095.99 | 1103 | 165.01±14.06^ab^ | 182.4±20.52^a^ | 158.07±10.88^b^ | 182.19±10.15^a^ | 95.89±4.99^c^ |
|  | (Methylthio)-benzene | 100-68-5 | 1095.42 | 1106 | 240.63±34.93^a^ | 195.08±19.58^c^ | 223.51±18.41^ab^ | 210.43±11.18^ab^ | 216.91±6.15^ab^ |
|  | Dodecanenitrile | 2437-25-4 | 1493.36 | 1490 | 127.26±16.43^a^ | 138.56±8.21^a^ | 46.68±5.52^c^ | 50.08±3.78^c^ | 69.73±2.36^b^ |

Note: ^a^ RI (DB), retention index of the compounds in samples, ^b^ RI (NIST), retention index of the compounds in samples in INST, ^c^ ND means the volatile compound was not detected in tea samples, ^d^ Different small letters in the same row indicated significant difference (*p* < 0.05)
